# Effects of Intracranial Pressure Monitoring on Mortality in Patients with Severe Traumatic Brain Injury: A Meta-Analysis

**DOI:** 10.1371/journal.pone.0168901

**Published:** 2016-12-28

**Authors:** Liang Shen, Zhuo Wang, Zhongzhou Su, Sheng Qiu, Jie Xu, Yue Zhou, Ai Yan, Rui Yin, Bin Lu, Xiaohu Nie, Shufa Zhao, Renfu Yan

**Affiliations:** 1 Department of Neurosurgery, Huzhou Central Hospital, Huzhou, Zhejiang, China; 2 Department of Medical College, Nursing College of Huzhou University, Huzhou, Zhejiang, China; University of South Florida, UNITED STATES

## Abstract

**Background:**

The Brain Trauma Foundation (BTF) guidelines published in 2007 suggest some indications for intracranial pressure (ICP) monitoring in severe traumatic brain injury (TBI). However, some studies had not shown clinical benefit in patients with severe TBI; several studies had even reported that ICP monitoring was associated with an increased mortality rate. The effect of ICP monitoring has remained controversial, regardless of the ICP monitoring guidelines. Here we performed a meta-analysis of published studies to assess the effects of ICP monitoring in patients with severe TBI.

**Methods:**

We searched three comprehensive databases, the Cochrane Library, PUBMED, and EMBASE, for studies without limitations published up to September 2015. Mortality, ICU LOS, and hospital LOS were analyzed with Review Manager software according to data from the included studies.

**Results:**

Eighteen eligible studies involving 25229 patients with severe TBI were included in our meta-analysis. The results indicated no significant reduction in the ICP monitored group in mortality (hospitalized before 2007), hospital mortality (hospitalized before 2007), mortality in randomized controlled trials. However, overall mortality, mortality (hospitalized after 2007), hospital mortality (hospitalized after 2007), mortality in observational studies (hospitalized after 2007), 2-week mortality, 6-month mortality, were reduced in ICP monitored group. Patients with an increased ICP were more likely to require ICP monitoring.

**Conclusion:**

Superior survival was observed in severe TBI patients with ICP monitoring since the third edition of “Guidelines for the Management of Severe Traumatic Brain Injury,” which included “Indications for intracranial pressure monitoring,” was published in 2007.

## Introduction

Traumatic brain injury (TBI), a huge clinical challenge and public health problem, is one of the leading causes of mortality and disability worldwide[1]. In the USA, TBI is evaluated in approximately 4.8 million emergency department visits per year, and 52% of evaluations have involvement with TBI [2]. The incidence of emergency department visits, hospital admissions, and deaths of TBI is 78.2%, 18.5%, and 3.3%, respectively[3]. Among TBI patients treated in hospital, severe TBI has an incidence of 5% nearly, and the intensive care unit (ICU) mortality rate is approximately 31% [4].

TBI management varies with increasing intracranial pressure (ICP). Mortality and disability in patients with TBI are the consequence of the primary injury, as well as secondary damage resulting from increasing ICP due to traumatic mass lesions, increasing hematoma, and brain edema or swelling. According to the ICP data from patients with TBI, doctors can calculate cerebral perfusion pressure (CPP) and assess worsening intracranial pathology to guide therapy [5]. One study showed that an ICP > 20 mmHg was strongly correlated with subsequent brain death [6]. Patients with severe TBI (Glasgow Coma Scale [GCS] score ≤ 8), especially those with an abnormal admission computed tomography (CT) scan, have the highest risk of intracranial hypertension (ICH) [7, 8]. Hence, both degree and duration of increased ICP in patients with severe TBI are closely related with poor outcomes [9, 10].

Increased ICP is the leading cause of death from head trauma in patients who reach the hospital alive. Patients who respond to ICP lowering therapies with subsequently improved CPP have an obvious better outcome than those who do not [11]. Besides, Brain Trauma Foundation (BTF) guidelines suggest some indications for ICP monitoring in severe TBI. but do not confirm the clinical benefit in patients with severe TBI. Indeed, several studies reported that ICP monitoring was associated with increased mortality and a longer length of stay (LOS). Is ICP monitoring, a way to guide the management of patients with severe TBI at high risk of ICH, improving patient prognosis? Several clinical studies demonstrated that ICP monitoring for TBI provided beneficial effects on decreasing mortality [12, 13]. However, many retrospective studies insisted on that ICP monitoring did not significantly improve mortality or Glasgow Outcome Scale score (GOS) [14] and even might increase mortality rates [15, 16]. Over the past decades, there not have drawn a clear conclusion on the effects of ICP monitoring of TBI patients. Before 2007, whether to take ICP monitoring depended on the intensive management experience. In 2007, the BTF guidelines recommend that ICP should be monitored in salvageable patients with severe TBI with an abnormal head CT or a normal CT scan plus at least two of the following risk factors at admission: age>40 years, unilateral or bilateral motor posturing, and systolic blood pressure<90 mmHg [17]. Did the survival rate improve in severe TBI patients with ICP monitored since the third edition of “Guidelines for the Management of Severe Traumatic Brain Injury” published in 2007, which included “Indications for intracranial pressure monitoring”? Therefore, we are eager to elucidate the role of ICP monitoring in the prognosis of patients with severe TBI. Additionally, whether ICP monitoring had a good effect on improving survival rate under the guild of guidelines published in 2007 remains to be further investigated. We then conducted a meta-analysis of the published literature to validate the value of ICP monitoring in patients with severe TBI.

## Materials and Methods

### Literature Search

In order to avoid controversial issues, we conducted a meta-analysis without a previous review protocol. We searched studies without language limitations published up to September 2015 by searching three databases, EMBASE, PUBMED, and the Cochrane Library. Using MeSH terms combined with free terms, including brain injury, head injury, traumatic brain injury, TBI, and intracranial pressure. The references of the identified studies were then screened for more relevant articles.

### Study Selection

The studies were screened using the following inclusion criteria according to the PICOS criteria (18) as follows: (1) *Participants* (P): All of the patients suffered from severe TBI (GCS≤8); (2) *Intervention* (I) and *Comparator* (C): ICP monitoring was considered the intervention, and we compared the prognosis of patients in the ICP monitoring group versus those in the no-ICP monitoring group; (3) *Outcomes* (O): Mortality rate was reviewed; and (4) *Study Design* (S): We reviewed case-control studies, cohort studies, and randomized controlled trials (RCTs).

We excluded studies for which: (1) information was not sufficient to extract the data or failed to retrieve data from the authors; (2) repeat published studies or data were available but a larger or more recent study existed; or (3) full text was not available.

### Data Extraction and Methodological Assessment

We extracted the following data independently from the included studies: the first author surname, publication year, country, study design, participant age, sample size, mortality, and study quality. The assessment of methodological quality of nonrandomized studies was performed by two reviewers using the Newcastle-Ottawa Scale (NOS) criteria with a range of 0–9 stars [18]. The study was deemed to have a relatively higher quality if it received≥6 stars. The methodology of including the RCT study type was evaluated by the Cochrane Risk of Bias Assessment tool [19]. All of the disagreements were discussed according to the standardized manner or resolved in consultation with the third author.

### Statistical Analysis

Review Manager Software (version 5.3, Cochrane Collaboration) was used for the statistical analysis. Relative risk (RR) values and the corresponding 95% confidence intervals (CI) were used to assess the value of the ICP monitor. Dichotomous variables were analyzed using RR for evaluating mortality, and 95% CI was calculated. Values of p<0.05 were considered statistically significant. Heterogeneity was tentatively divided into absent heterogeneity and notable heterogeneity. The I^2^ test and Chi-square test were adopted to assess heterogeneity. Values of I^2^<50% and p>0.1 indicated lower inter-study variation [20, 21].If the heterogeneity was notable, the random effects model was employed for the analysis; otherwise, the fixed effect model was adopted [22]. In those studies with notable heterogeneity, subgroup analyses were conducted to explore the potential factors of heterogeneity. Sensitivity analyses were performed to investigate the stability of each study for the overall estimation by excluding each one by one. Moreover, publication bias analyses were conducted to avoid the exaggerated effect of the overall treatment estimates [23].

## Results

### Study Characteristics

**Fig 1:**
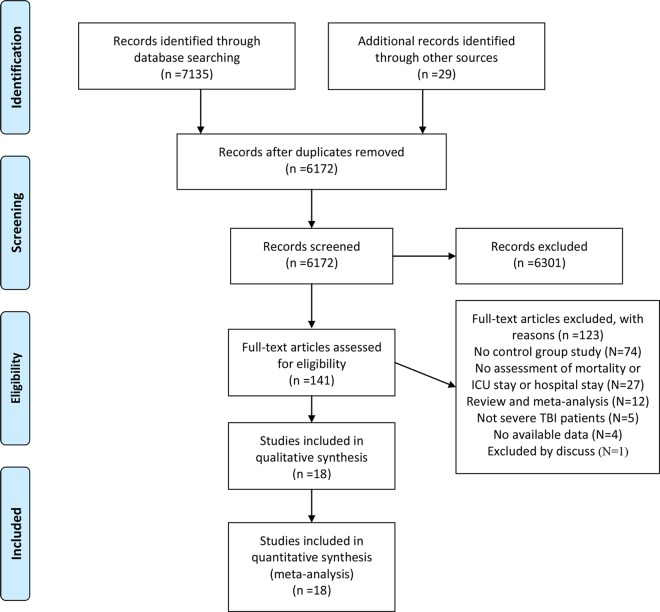
Flow diagram of literature search and selection process.

An initial search of three databases yielded 5172 studies, of which 141 articles required further screening consisting of reading the title and abstract. Most studies in Table 1 had a crude RR around 1, but the RR according to the data from one observational study [24] was 9.8, suggesting that patients who received ICP monitoring had an almost 10 times higher mortality, which seemed clinically impossible. This study might be a huge potential selection bias. Finally, we excluded it by a serious discussion. Ultimately, 18 of these articles met the inclusion criteria and were used to assess the value of ICP monitoring in patients with severe TBI [12, 13, 15, 16, 25–38] (Fig 1). Of 16 observational studies and two RCTs, nine studies were conducted in America, five in Asia, three in Europe, and one in Africa. Ten studies described the effect of ICP monitoring inclusion after 2007. Four studies contained detailed information about ICU mortality, nine on hospital mortality, three on 2-week mortality, one on 4-week mortality, and three on 6-month mortality. The quality of 16 observational studies was relatively higher (Table 1). The included RCTs quality was assessed according to the Cochrane Risk of Bias Assessment tool. Two RCTs have unclear risk on selection bias (Allocation concealment), detection bias, reporting bias and other bias, low risk on attrition bias; One study [31] has a low risk on selection bias (Random sequence generation), while the other has a high risk [29]. More information about the included studies is shown in Table 1.

**Table 1:** Characteristics of studies included in the meta-analysis.

Study and Year	Country	Design	Study Period	Sample Size	Age (year, mean or mean±SD)	Risk of Mortality (RR, 95%CI)	Study Quality
Mauritz 2007	Austria	Multicenter observational study	__	415	50±21	0.89(0.67–1.20) hospital mortality	7
Shafi 2008	USA	Multicenter observational study	1994–2001	1646	33±0.3	1.75(1.40–2.19) hospital mortality	8
Mauritz 2008	Austria	Multicenter observational study	1998–2004	1856	46^a^ 53^b^	1.03(0.91–1.17) hospital mortality	8
Griesdale 2010	USA	Single center observational study	2000–2006	171	35±15.4^a^ 42±18.0^b^	2.32(1.17–4.61) hospital mortality; 1.82(0.89–3.72) 4-week mortality	8
Haddad 2011	Saudi Arabia	Single center observational study	2001–2008	477	28.0±11.3^a^ 29.3±14.9^b^	1.17(0.56–2.44) ICU mortality; 1.38(0.78–2.45) hospital mortality	8
Kostic 2011	Srbiga	RCTs	2008–2010	61	40.98±21.70^a^ 43.41±22.31^b^	0.72(0.45–1.13)mortality	__
Biersteker 2012	Netherlands	Multicenter observational study	2008–2009	265	44a 53^b^	0.75(0.30–1.91) 6-month mortality	8
Chesnut 2012	USA	RCTs	2008–2011	324	29	0.70(0.48–1.03) 2-week mortality; 0.89(0.68–1.17) 6-month mortality	__
Farahvar 2012	USA	Multicenter observational study	2000–2009	1307	45.9±21.8^a^ 38.3±17.5^b^	0.59(0.47–0.74) 2-week mortality	8
Alali 2013	Canada	Multicenter observational study	2009–2011	10628	43a 53^b^	0.87(0.81–0.94) hospital mortality	8
Gao 2012	China	Single center observational study	2003–2009	36	>20	1.25(0.40–3.91)ICU/hospital mortality	7
Talving 2013	USA	Multicenter observational study	2010–2011	216	40.1±1.9^a^ 48.0±2.4^b^	0.61(0.44–0.84) hospital mortality	8
Alkhoury 2014	USA	Multicenter observational study	2001–2006	3107	8.5±5.9^a^ 8.4±6.0^b^	0.88(0.72–1.07) mortality	8
Kim 2014	Korea	Single center observational study	2010–2012	78	42.9±16.65^a^ 45.2±18.24^b^	0.47(0.22–0.99) 2-week mortality	8
Agrawal 2015	India	Multicenter observational study	2010-2-12	1345	31	0.57(0.48–0.68) hospital mortality	8
Alali 2015	Canada	Multicenter observational study	2010–2012	1705	9^a^ 8^b^	0.74(0.52–1.06) hospital mortality	8
Dawes 2015	USA	Multicenter observational study	2009–2010	822	>1	0.67(0.56–0.81) hospital mortality	8
Yuan 2015	China	Multicenter observational study	2012–2013	1077	47.76 ±16.03^a^ 48.04 ±17.71^b^	0.80(0.65–1.00) 6-month mortality	8

RCTs, Randomized Controlled Trials; a, Case Group; b, Control Group

### Risk of Mortality

**Fig 2:**
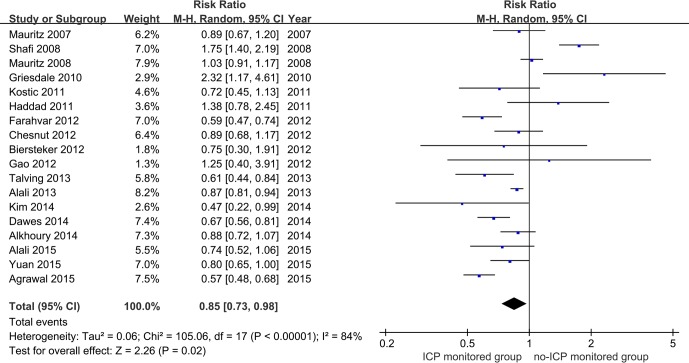
Effect of ICP monitoring on overall mortality among patients with severe TBI.

**Fig 3:**
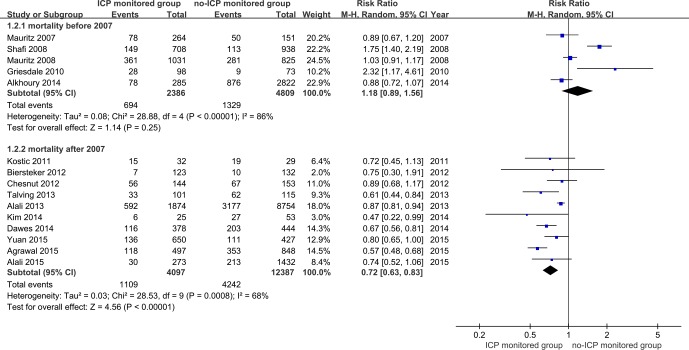
Forest plot of association between ICP monitoring and mortality in patients with severe TBI stratified by hospitalized time.

Eighteen studies containing 7637 patients with severe TBI who were subjected to ICP monitoring and 17862 with severe TBI who were not subjected to ICP monitoring were ultimately analyzed in this meta-analysis[12, 13, 15, 16, 24–38]. Fig 2 shows a forest plot comparing the ICP monitored and no-ICP monitored groups. The results indicated that ICP monitoring decreased the mortality rate of patients with severe TBI. The pooled RR for ICP monitoring was 0.85 (95% CI = 0.73–0.98, p<0.05; I^2^ = 84%, p for heterogeneity<0.1). We conducted subgroup analysis depending on time of hospital admission, study design and mortality rate classification, in order to explore the potential factors of heterogeneity. In the subgroup analysis, studies were divided into two groups according to time of hospital admission. Five studies[15, 25–27, 35] reported on the ICP embedded absolutely before 2007, while 10 studies examined the same after 2007 [12, 13, 29–32, 34, 36–38]. The subgroup provided efficient evidence that ICP monitoring reduced the mortality rate after 2007 (pooled RR = 0.72, 95% CI = 0.63–0.83, p<0.01; I^2^ = 68%, p for heterogeneity<0.1) but not before 2007 (pooled RR = 1.18, 95% CI = 0.89–1.56, p = 0.25; I^2^ = 86%, p for heterogeneity<0.1) (Fig 3).

**Fig 4:**
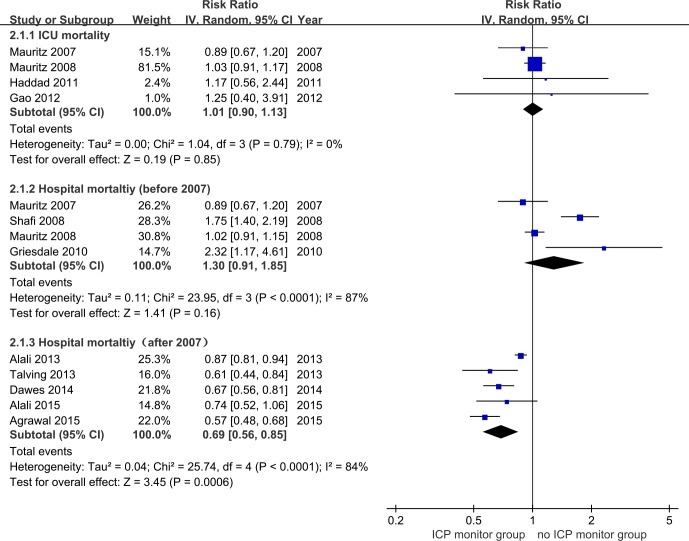
Forest plot of association between ICP monitoring and mortality in ICU mortality and in-hospital mortality.

Four studies [25, 26, 28, 33] provided available data showing no decreased risk of ICU mortality in the ICP monitor group (RR = 1.01, 95% CI = 0.90–1.13, p>0.05; I^2^ = 0%, p for heterogeneity>0.1) (Fig 4). Four studies [15, 25–27] (hospitalized before 2007) with 4088 patients and five studies [13, 32, 34, 37, 38] (hospitalized after 2007) with 14716 patients attached importance to analyzing the link between ICP monitoring and the risk of mortality. ICP monitoring was associated with a 31% reduction in hospital mortality of patients who were hospitalized after 2007 (RR = 0.69, 95% CI = 0.56–0.85, p<0.01; I^2^ = 84%, p for heterogeneity<0. 1), however, no significant result was obtained in patients hospitalized before 2007 (RR = 1.30, 95% CI = 0.91–1.85, p = 0.16; I^2^ = 87%, p for heterogeneity<0.1) (Fig 4).

**Fig 5:**
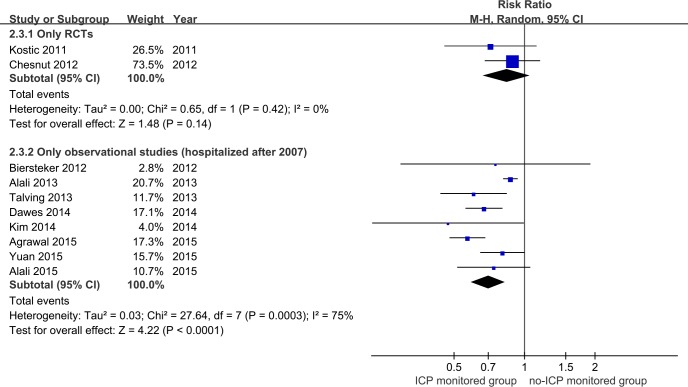
Forest plot of association between ICP monitoring and mortality in only RCTs, observational studies, and child populations.

As shown in Fig 5, the difference in mortality between the two RCTs [29, 31] did not differ significantly between the ICP monitor and no-ICP monitored groups (RR = 0.84, 95% CI = 0.66–1.06, p>0.05, I^2^ = 0, p for heterogeneity>0.1). However, in observational studies [12, 13, 30, 32, 34, 36–38], the mortality rate was reduced 30% in hospitalized patients with severe TBI after 2007 (RR = 0.70, 95% CI = 0.59–0.83, p<0.01; I^2^ = 75%, p for heterogeneity<0.1).

**Fig 6:**
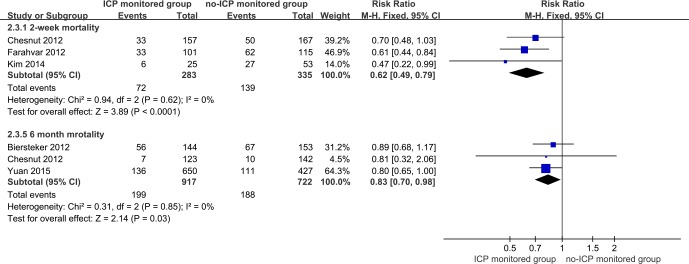
Forest plot of association between ICP monitoring and mortality expressed as 2-week and 6-month mortality rates.

Three studies contained estimated 2-week [16, 31, 36] and 6-month [12, 30, 31] mortality. Synthesized results indicated that both 2-week and 6-month mortality rates were greatly reduced in the ICP monitoring group (RR = 0.62, 95% CI = 0.49–0.79, p<0.01; I^2^ = 0%, p for heterogeneity>0.1; RR = 0.83, 95% CI = 0.71–0.98, p<0.05; I^2^ = 0%, p for heterogeneity>0.1, respectively) (Fig 6).

### Notable Heterogeneity

The included studies had notable heterogeneity in overall mortality rates, and the subgroup analyses did not eliminate partial heterogeneity according to hospital mortality before 2007, hospital mortality after 2007, observational studies only, and so on. The heterogeneity occurring in the studies was probably attributable to its local state of healthcare, clinical intervention inconsistencies, or other potential factors.

### Sensitivity Analysis and Publication Bias

**Fig 7:**
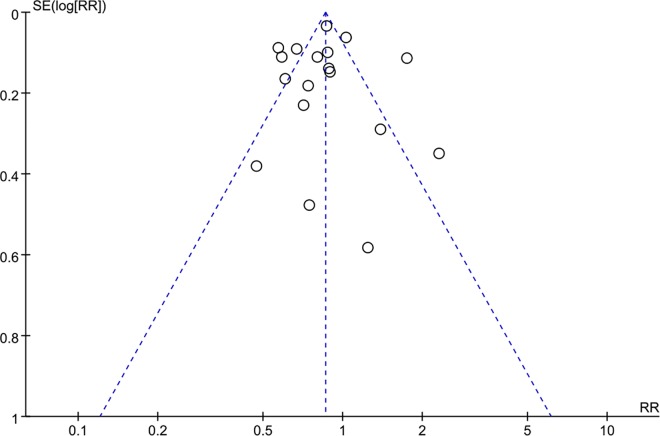
Funnel plot of publication bias of all included studies of mortality.

To ensure results for accuracy, we conducted a sensitivity analysis by omitting each study in turn to investigate the influence of each on the final risk assessment. The results showed a lack of stable influence on mortality in all included studies but a stable influence in studies whose patients were hospitalized after 2007. No apparent publication bias was found on visual examination of the funnel plots (Fig 7). However, this form of publication bias assessing lacks of reliability due to the limitation of Review Manager Software.

## Discussion

To our knowledge, this meta-analysis of published clinical studies is the first separated analyses performed for different time points of mortality and RCTs vs observational studies to discuss the association between mortality risk and ICP monitoring in patients with severe TBI. The results of our meta-analysis imply a decreased mortality risk for overall mortality, mortality (hospitalized after 2007), 2-week mortality, 6-month mortality and hospital mortality (hospitalized after 2007), whereas the ICU and hospital LOS were prolonged in the ICP monitoring group.

Several decades ago, Narayan et al reported that patients with severe TBI were at higher risk of developing ICH, especially for those with an abnormal CT scan [8]. A large number of studies then confirmed that outcomes tended to be significantly improved in those TBI patients if their ICP<20 mmHg or in those who responded to ICP lowering treatment [39–41]. According to clinical experience, reduced cerebral perfusion contributed to poor outcomes and a minimum cerebral perfusion pressure (CPP) of 50mmHg had been advocated in the Lund concept which was a theoretical approach to the treatment of severe TBI [42]. CPP incorporated with mean arterial blood pressure and ICP parameters was an indirect measure of cerebral perfusion. Thus, there might be a reduction in mortality with ICP monitoring under the guide of ICP monitoring of ICH treatment. Nevertheless, even though an extensive literature has been published, the role of invasive ICP monitoring remains conflicting. Most published observational studies did not support the use of ICP monitoring in patients with severe TIB. Su et al performed a meta-analysis of patients with TBI and implied that no benefit was achieved in patients who underwent ICP monitoring [43], and Yuan implicated that ICP monitoring did not contribute to lower mortality rates in patients with severe TBI [44]. However, the results of the above meta-analyses did not conform to the theoretical expectations of ICP monitoring. Therefore, we searched for more published studies and conducted a second meta-analysis. Our results testified that ICP monitoring decreased the mortality of patients with severe TBI. Nevertheless, the results of sensitivity analysis, risk assessment between ICP monitoring and overall mortality were lack of stability. Further studies focused on the effect of ICP monitoring on survival rate in patients with TBI longing are warranted

The third edition of the “Guidelines for the Management of Severe Traumatic Brain Injury,” which introduced the “Indications for intracranial pressure monitoring” was updated by the BTF in 2007 [17]. Before 2007, ICP monitoring indication was not unified. ICP monitoring was adopted totally based on the individual clinical experience and relevant suggestions. Some patients were suggested to accept ICP monitoring since the specialized indications had been revised and published in 2007. In the most of included studies, the TBI patients hospitalized after 2007 and met the indications for ICP monitoring. Hence we made a decision to split the sample at the time point of 2007 based on the changes of guidelines, and the decision did make sense. Patients admitted to the hospital after 2007 who were subjected to ICP monitoring had a better prognosis with a 28% reduction in total mortality rate and a 31% reduction in hospital mortality rate. Synthesized results suggested a mortality benefit with ICP monitoring (hospitalized after 2007). Yet no similar result was observed in hospital mortality (hospitalized before 2007). Unfortunately, ICP monitoring did not effectively decrease the mortality in ICU setting. However, the population of all the included studies involving ICU mortality was hospitalized before 2007. The discrepancy in mortality (pre and post 2007) probably results from updated TBI guidelines. The indications for ICP monitoring from TBI guideline lead a standardized treatment approaches and might exert significant improvements.

A subgroup analysis of study design was performed to reduce methodological heterogeneity. Chesnut et al conducted a RCT in which they attached care to maintaining an ICP≤20 mmHg and found no more benefit than the imaging and clinical examination group [31]. Synthesized result of two RCTs showed no insight reduction on mortality rate of ICP monitored group, but the conclusions did not statistically significant (p>0.05). In eight observational studies, patients (hospitalized after 2007) subjected to ICP monitoring had a longer survival. The inter-study design differences in the individual therapies reflected differences in treatment approaches. The survival rate was not associated with study design, so the small population of RCTs and the limitations of observational studies should be considered. Besides, Benchmark Evidence from South American Trials: Treatment of Intracranial Pressure (BEST TRIP) trial stated that the lack of efficacy might be attributable to some factors, such as different approaches to management, toxic effects of the therapeutic agents [31]. Furthermore, BTF guidelines recommended that the ICP-reducing therapy should be initiated when ICP threshold is above 20 mmHg [45]. However, we found that the initial ICP of most patients (63%) included in BEST TRIP trial was below 20 mmHg. Considering the above reasons, we come to the thought with caution that ICP monitoring might provide a better prognosis for severe TBI patients.

In our meta-analysis, the mortality of ICP monitored group (hospitalized after 2007) was improved, as well as 2-week and 6-month mortality rates. Also, the fourth edition of guidelines updated the monitoring recommendation that ICP monitoring reduced in-hospital and 2-week post-injury mortality [46]. However, results of 2-week and 6-month mortality rates implied that an increased sample size was required to enhance reliability of the conclusion. In addition, long-term functional outcomes were not investigated in this meta-analysis due to insufficient data of included studies. Several studies come to the findings that no distinct difference had been found in the unfavorable outcomes at the time point of 6-month difference between ICP monitored group and no ICP monitored group [12, 37]. It is noteworthy that various studies considered that an ICP monitor might lead to a poor prognosis compared to patients subjected to medical management only [15, 24, 27]. Shafi et al firmly believed that ICP monitoring with worsening survival should be blamed on misapplied interventions designed to reduce ICP [15], for example, hyperventilation, which was initially considered to decrease cerebral perfusion yet might reversely cause cerebral ischemia [47, 48].

According to the Cochrane Handbook Systematic Reviews, there are three forms of heterogeneity, statistical, methodological, and clinical. Subgroup analyses were conducted to reduce higher heterogeneity, which might drive the results almost entirely using separated time point (pre 2007/after 2007), different mortality measurements, different study designs, yet no obvious reduction was observed. Almost all the severe TBI results from violent injury, but also subjects to trunk trauma, shock, or other fatal factors, so isolated severe TBI with ICP and no-ICP monitoring treatments in included studies are wanted to reduce heterogeneity. Besides, patients with severe TBI in ICU required various treatments, this may contribute to huge heterogeneity. Taking ICP monitoring as the independence of intervention measure to assess the effect of ICP in isolated severe TBI is essential, however, in fact, it is very difficult to achieve this. In total, according to the current clinical evidence, ICP monitoring in severe TBI warrants further confirmations.

Several limitations of this meta-analysis should be considered. First of all, most of the included studies are observational studies, making it impossible to ensure the independences of intervention measures. Another limitation is that mortality data seem to be a lack of consistency since they has included ICU mortality, hospital mortality, and 2-week and 6-month mortality, while we pooled all these data together for the meta-analysis, a more detailed classification should be considered in future analyses. Moreover, the data of mortality do not contain the long-term functional outcome, thus, the effects of ICP monitoring may lack of comprehensive assessment. Additionally, the included studies without a single management plan and analogous study designs produce apparent methodologically heterogeneity, which may result in unstable results. Finally, isolated severe TBI with ICP and no-ICP monitoring treatments should be picked out to analyze, thus the independence of the intervention measure may help to draw a significant conclusion.

## Conclusion

Superior survival was observed in severe TBI patients with ICP monitoring, yet the role of ICP monitoring in severe TBI patients remain to be further elucidated, more rigorously designed studies with long-term follow-up on the effects of ICP monitoring are needed.

## Supporting Information

S1 File PRISMA 2009 Checklist. (DOC)

S2 File Search Strategy. (DOCX)
