# Supplementary material for: Effects of Intracranial Pressure Monitoring on Mortality in Patients with Severe Traumatic Brain Injury: A Meta-Analysis
Source: PLoS One. 2016 Dec 28;11(12):e0168901. doi: 10.1371/journal.pone.0168901 (PMC5193438; doi:10.1371/journal.pone.0168901)
Supplement: S2 File — (DOCX) [file pone.0168901.s002.docx]

**Search Stragety**

#1 "brain injuries"[Mesh]

#2 "brain Injuries"

#3 "traumatic brain injury"

#4 TBI

#5 "cerebral trauma"

#6 "brain trauma"

#7 "head injury"

#8 #1 OR #2 OR #3 OR #4 OR #5 OR #6 OR #7

#9 "intracranial pressures"[Mesh]

#10 "intracranial pressures"

#11 "intracranial pressures monitor*"

#12 ICP

#13 "pressures, intracranial"

#14 "intracranial hypertension"

#15 #9 OR #10 OR #11 OR #12 OR #13 OR #14

#16 #8 And #15
